# Contact Resonance Atomic Force Microscopy Using Long, Massive Tips

**DOI:** 10.3390/s19224990

**Published:** 2019-11-15

**Authors:** Tony Jaquez-Moreno, Matteo Aureli, Ryan C. Tung

**Affiliations:** Mechanical Engineering Department, University of Nevada, Reno, 1664 N. Virginia St, Reno, NV 89557-0312, USA; tonyj@nevada.unr.edu (T.J.-M.); maureli@unr.edu (M.A.)

**Keywords:** cantilever based sensors, atomic force microscopy, contact resonance, trolling mode

## Abstract

In this work, we present a new theoretical model for use in contact resonance atomic force microscopy. This model incorporates the effects of a long, massive sensing tip and is especially useful to interpret operation in the so-called trolling mode. The model is based on traditional Euler–Bernoulli beam theory, whereby the effect of the tip as well as of the sample in contact, modeled as an elastic substrate, are captured by appropriate boundary conditions. A novel interpretation of the flexural and torsional modes of vibration of the cantilever, when not in contact with the sample, is used to estimate the inertia properties of the long, massive tip. Using this information, sample elastic properties are then estimated from the in-contact resonance frequencies of the system. The predictive capability of the proposed model is verified via finite element analysis. Different combinations of cantilever geometry, tip geometry, and sample stiffness are investigated. The model’s accurate predictive ranges are discussed and shown to outperform those of other popular models currently used in contact resonance atomic force microscopy.

## 1. Introduction

Contact Resonance (CR) atomic force microscopy (AFM) is a relatively new, popular measurement technique used to characterize nanoscale material properties. CR AFM relies on analyzing the coupled vibrations of an AFM cantilever probe that is resonated while in permanent, net-repulsive contact with a sample of interest. CR AFM has been used to characterize the properties of thin metallic films [1] and polymer blends [2]. CR AFM has also been used to measure the viscoelastic loss tangents of polymer blends [3], study the effect of relative humidity on the viscoelastic properties of organic thin films [4], and conduct photorheological measurements to study curing kinetics of polymers [5]. Additionally, CR AFM has been used to measure buried, subsurface nanostructures [6,7,8,9] that are not visible in typical topographic AFM measurements. Finally, the principles of contact resonance have been used to enhance other popular modes of AFM, such as electrochemical strain microscopy (ESM) [10,11,12] and piezoresponse force microscopy [13,14] (PFM), and researchers have developed new experimental measurement procedures and techniques for CR AFM that aim to increase the accuracy of these coupled methods [15].

The underlying theoretical model of CR AFM utilizes the Euler–Bernoulli (EB) beam model. To date, researchers have included the effects of tip offset [16], tip height effects [17], normal and lateral contact springs [17], Poisson’s ratio of the sample material [18], and sample viscoelasticity effects [19]. More recent modeling efforts have included the effect of using U-shaped cantilever probes [20] and using a Timoshenko beam model in the theoretical framework [21].

Recently, AFM cantilever sensor designs have included large sensing tips. For example, the qPlus sensor [22] uses a massive tip affixed to a quartz tuning fork tine and is capable of conducting extremely sensitive measurements. Long, massive tips have been affixed to AFM cantilevers and are used in operational modes such as “Trolling Mode” [23,24] to measure material properties of polymers and living cells. By using long sensing tips, researchers are able to remove the main cantilever body from liquid environments [25,26,27], thereby reducing unwanted hydrodynamic forces on the cantilever [28,29,30,31,32,33,34] and reducing extraneous noise sources prevalent in liquid AFM imaging environments [35]. Notwithstanding the practical importance that these techniques are gaining and their potential to open up new sensing modalities in CR AFM, rigorous analyses of the effect that the long, massive tip has on the system dynamics are so far lacking in the established literature. Therefore, incomplete understanding of the behavior and idiosyncrasies of cantilever-based sensors endowed with long, massive tips is limiting their applications and adoption in key sensing areas.

To bridge this knowledge gap, in this work, we analyze the behavior of AFM cantilever probes with long, massive tips to determine their effect on the surface-coupled vibrations of the system. To this aim, we modify the traditional EB model for cantilever vibration with a new set of boundary conditions that models both the presence of a long, massive tip (via the transverse force and moment that the tip, modeled as a rigid body, exerts on the cantilever) and contact with an elastic sample. Since the effect of the tip is only included in the boundary conditions, possible dynamics of the tip are not explicitly captured. However, a procedure to estimate the effective inertia and moment of inertia of the tip, as seen by the cantilever, is proposed based on a novel interpretation of flexural and torsional modes of vibration of the structure when not in contact with the sample. Contact with an elastic sample is modeled via an orthogonal set of springs, coupled to the cantilever tip, capable of linear elastic response in the transverse and in-plane directions.

An estimation procedure for the sample stiffness is then proposed based on analysis of the free and in-contact resonance frequencies of the system. The dynamics of the system are also investigated using a finite element model simulation to verify the proposed model and assess the impact of the modeling hypotheses. Particular interest is placed on the flexibility of the tip and its effect on the accuracy of the prediction. The proposed model is shown to be superior to traditional models which ignore inertia and moment of inertia of the long, massive tip for a broad range of system dimensions and stiffness parameters. Thus, the proposed model paves the way for correct interpretation of trolling mode CR AFM experiments.

The remainder of the paper is organized as follows. In Section 2, we develop the theoretical model for flexural and torsional vibrations and introduce the characteristic equations on which the estimation procedure hinges. In Section 3, we detail our numerical experiments conducted in lieu of physical experiments on fabricated cantilever sensors. Results and discussions are presented in Section 4, where we discuss the limits of applicability of the proposed model. Conclusions are reported in Section 5.

## 2. Theory and Model Development

**Figure 1:**
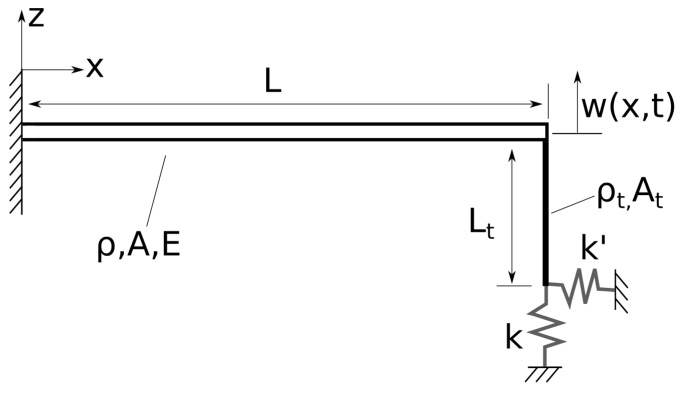
Euler–Bernoulli beam model of a cantilever with a long, massive tip in contact with an elastic substrate. Here, the tip is assumed to be rigid. The substrate is modeled through linear springs.

In this section, we develop a simple model for a cantilever beam endowed with a long, massive tip in contact with an elastic substrate, representative of typical CR AFM configurations in trolling mode operations. To maintain a realistic model, with manageable complexity, we introduce a set of assumptions whose validity will be analyzed in the rest of the paper. Figure 1 depicts a schematic representation of the idealized system under study. Small amplitude vibrations are considered throughout.

### 2.1. The Flexural Problem

With reference to Figure 1, we first focus on flexural vibrations of the beam in the xz-plane, exclusively. The beam is assumed to be of an isotropic and homogeneous material, with Young’s modulus *E* and Poisson’s ratio ν. Furthermore, ρ denotes the mass density (per unit volume) of the beam; *A* is the rectangular cross-sectional area, assumed to be constant along the axis; and *L* is the length of the beam. In this model, the long sensing tip is fixed at the end of the beam, with length $L_{t}$, mass density $\rho_{t}$, and circular cross-sectional area $A_{t}$. At this stage, the tip is assumed to be rigid, and connected to two one-dimensional linear springs of constants *k* and $k^{\prime }$ in the *z*- and *x*-directions, respectively. These springs model the normal and lateral stiffness of the sample in contact. The equations of motion for the transverse vibrations of the unforced system are given by [36]

(1) $$\rho A\frac{\partial^{2}w\left(x,t\right)}{\partial t^{2}}+EI\frac{\partial^{4}w\left(x,t\right)}{\partial x^{4}}=0,$$

where *I* is the second area moment of inertia of the cantilever beam and w(x,t) represents the transverse displacement of the beam at a given location *x* along the axis and a specified time *t*.

Translational and rotational inertia effects of the massive tip, along with sample stiffness, are incorporated into the model in Equation (1) via the following boundary conditions [36]:

(2a) $$\begin{array}{cc} \; & w(0,t)=0, \end{array}$$

(2b) $$\begin{array}{cc} \; & \frac{\partial w}{\partial x}\left(0,t\right)=0, \end{array}$$

(2c) $$\begin{array}{cc} EI & \frac{\partial^{2}w\left(L,t\right)}{\partial x^{2}}=-I_{t}\frac{\partial^{3}w\left(L,t\right)}{\partial x\partial t^{2}}-k^{\prime }L_{t}^{2}\frac{\partial w(L,t)}{\partial x}, \end{array}$$

(2d) $$\begin{array}{cc} EI & \frac{\partial^{3}w\left(L,t\right)}{\partial x^{3}}=m_{t}\frac{\partial^{2}w\left(L,t\right)}{\partial t^{2}}+kw\left(L,t\right), \end{array}$$

where $I_{t}$ is the rotational inertia and $m_{t}$ is the total mass of the sensing tip. In Equations (2c) and (2d), the sensing tip has effectively been modeled as a point-mass and point-inertia. Specifically, in Equation (2c), the cantilever end is subject to a bending moment due to the rotational inertia of the tip, along with the reaction from the lateral stiffness of the sample. Similarly, in Equation (2d), the cantilever end is subject to a shear force due to the translational inertia of the massive tip and to the normal stiffness of the sample. Note that, consistent with the assumptions of small displacements and deformations, higher order contributions to tip shear force and bending moment due to changes in length of the cantilever are neglected. It is also important to observe that any effects that may be related to deformability of the tip are ignored.

For free vibrations at a given frequency ω, the boundary condition in Equation (2c) is equivalent to the effect of a torsional spring connected to the cantilever tip, with effective torsional (dynamic) stiffness given by $K_{T}=k^{\prime }L_{t}^{2}-\omega^{2}I_{t}$. Similarly, the boundary condition in Equation (2d) is equivalent to the effect of a normal spring connected to the cantilever tip, with effective (dynamic) stiffness given by $K_{N}=k-\omega^{2}m_{t}$. These effective dynamic stiffnesses will be used later in the discussion of the model’s performance.

Through dimensional analysis of the equations of motion and associated boundary conditions, we identify the following governing nondimensional parameters: α is the nondimensional ratio of the normal spring stiffness *k* to the cantilever static stiffness $k_{c}=\left(3EI\right)/L^{3}$, so that $\alpha =k/k_{c}$; Δ is the nondimensional tip mass given by $\Delta =m_{t}/\left(\rho AL\right)$; ${\hat{I}}_{t}$ is the nondimensional rotational inertia of the tip given by ${\hat{I}}_{t}=I_{t}/\left(\rho AL^{3}\right)$; ϕ is the ratio of the lateral to the normal spring stiffnesses $\phi =k^{\prime }/k$; and $\ell =L_{t}/L$ is the ratio between the tip length and cantilever length. The limit of α=0 corresponds to the case of an “unsprung” cantilever, and the limit of $\Delta ={\hat{I}}_{t}=0$ corresponds to the case of an ideally massless tip.

Next, as in standard practice [36], we assume that the solution for w(x,t) is separable, that is w(x,t)=W(x)T(t). Substituting this ansatz into Equation (1) results in a fourth order ordinary differential equation (ODE) in the spatial dimension *x* and a second order ODE in the time dimension *t*. The general form of the spatial solution is given by $W\left(x\right)=C_{1}\cos\left(\lambda x\right)+C_{2}\sin\left(\lambda x\right)+C_{3}\cosh\left(\lambda x\right)+C_{4}\sinh\left(\lambda x\right)$, where λ is the separation constant. The general spatial solution along with the boundary conditions in Equation (2) form the eigenvalue problem (EVP) that governs the eigenmodes and eigenfrequencies of the system. Solution of the EVP generates the characteristic equation $f(\lambda L,\alpha ,\Delta ,{\hat{I}}_{t},\phi ,\ell )=0$, which describes the relationship between the natural frequencies of the system and the governing nondimensional parameters. Here, λL are the countably infinite nondimensional natural frequencies of the system given by ${\left(\lambda L\right)}^{4}=\omega^{2}\left(\rho AL^{4}\right)/\left(EI\right)$, where ω is the dimensional natural frequency. The complete characteristic equation for transverse vibrations of the system is given by

(3) $$\begin{array}{c} {}[\left(-2\Delta {\hat{I}}_{t}{\left(\lambda L\right)}^{8}+\left(2+\left(6\Delta \ell^{2}\phi +6{\hat{I}}_{t}\right)\alpha \right){\left(\lambda L\right)}^{4}-18\ell^{2}\alpha^{2}\phi \right)\cos\left(\lambda L\right)\\ +6\left(\lambda L\right)\sin\left(\lambda L\right)\left(-{\hat{I}}_{t}{\left(\lambda L\right)}^{6}/3+\phi \ell^{2}\alpha {\left(\lambda L\right)}^{2}-\Delta {\left(\lambda L\right)}^{4}/3+\alpha \right)]\cosh\left(\lambda L\right)+\\ 6\left(\lambda L\right)\left(-{\hat{I}}_{t}{\left(\lambda L\right)}^{6}/3+\phi \ell^{2}\alpha {\left(\lambda L\right)}^{2}+\Delta {\left(\lambda L\right)}^{4}/3-\alpha \right)\sinh\left(\lambda L\right)\cos\left(\lambda L\right)+2\Delta {\hat{I}}_{t}{\left(\lambda L\right)}^{8}+\\ \left(2+(-6\Delta \ell^{2}\phi -6{\hat{I}}_{t})\alpha \right){\left(\lambda L\right)}^{4}+18\ell^{2}\alpha^{2}\phi =0. \end{array}$$

Equation (3) defines the relationship between the transverse natural frequencies of vibration of the system, the sample stiffness in both the normal and lateral directions, and the tip mass and rotational inertia.

### 2.2. The Torsional Problem

In the development of the model, we use the freely vibrating, unsprung torsional modes of vibration of the system to estimate the rotational inertia of the massive tip. Within this approach, we continue to assume that the tip is rigid. Note that, because of its circular cross section, the tip is symmetric about the axes of rotation excited in transverse and torsional bending motions. This means that the tip rotational inertia identified from torsional oscillation can reasonably be used as a proxy for the tip rotational inertia needed in Equation (2) and, thus, in Equation (3).

With reference to the schematics in Figure 1, and focusing exclusively on torsional vibrations about the *x*-axis of the beam, the equations of torsional motion of the free, unsprung system are given by [36]

(4) $$\rho J\frac{\partial^{2}\theta \left(x,t\right)}{\partial t^{2}}=C\frac{\partial^{2}\theta \left(x,t\right)}{\partial x^{2}},$$

where *J* is the polar moment of inertia of the beam cross section, θ(x,t) is the twist angle of the beam, and *C* is the torsional rigidity of the beam. For a rectangular cross section of thickness *h* and width *b*, *C* is given by $C=\kappa Gh^{3}b$, where κ is given by [37]

(5) $$\kappa =\frac{1}{3}\left(1-\frac{192}{\pi^{5}}\frac{h}{b}\sum_{i=1,3,5,\ldots }^{\infty }\frac{1}{i}\tanh\left(i\pi \frac{b}{h}\right)\right)$$

and G=E/[2(1+ν)] is the shear modulus of the beam. For thin cross sections with h≪b, κ is well approximated by the value 1/3, see for example [34]. The boundary conditions for Equation (4) are given by

(6a) $$\begin{array}{cc} \; & \theta (0,t)=0, \end{array}$$

(6b) $$\begin{array}{cc} C & \frac{\partial \theta (L,t)}{\partial x}=-I_{t}\frac{\partial^{2}\theta \left(L,t\right)}{\partial t^{2}}, \end{array}$$

which show that the free end of the cantilever is subject to a twisting torque caused by the rotational inertia of the tip. Equation (6b) suggests the existence of an additional nondimensional parameter, namely, ${\hat{I}}_{tor}=I_{t}/\left(\rho JL\right)$, which represents the nondimensional rotational inertia of the tip.

By assuming a separable solution for θ(x,t), we obtain the following characteristic equation

(7) $$\left(\beta L\right)\cot\left(\beta L\right)-{\hat{I}}_{tor}{\left(\beta L\right)}^{2}=0,$$

where βL are the nondimensional natural frequencies of torsional vibration given by $\beta L=\omega_{tor}\sqrt{\rho J/C}L$, and $\omega_{tor}$ are the dimensional natural frequencies of torsional vibration. Equation (7), in the symbolic form $g(\beta L,{\hat{I}}_{tor})=0$, defines the relationship between the freely vibrating torsional modes and the rotational inertia of the massive tip.

### 2.3. Sample Stiffness Identification Procedure

**Figure 2:**
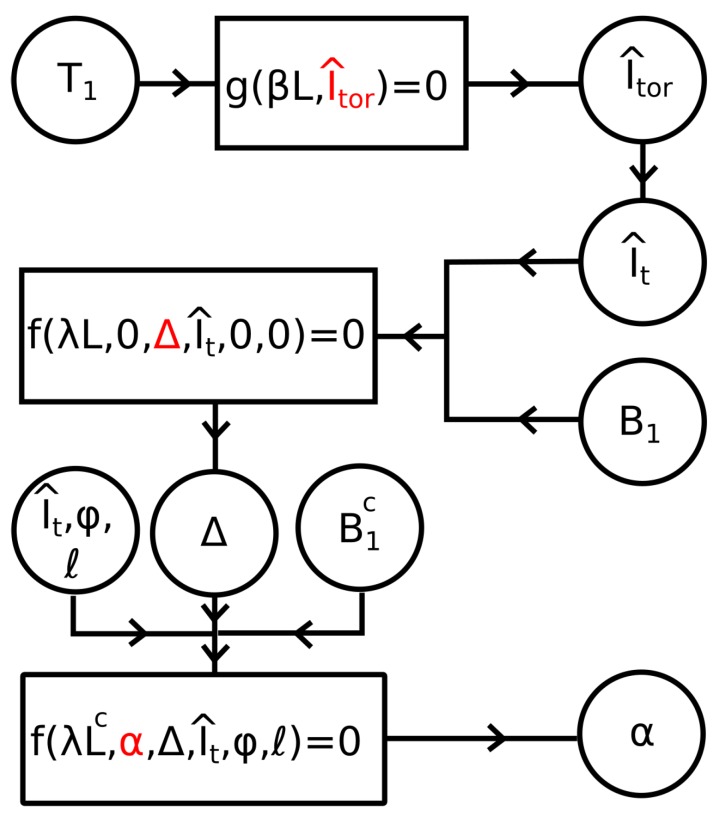
Schematic flowchart of the estimation procedure. At each step, the quantities highlighted in red are the unknowns to be estimated.

Figure 2 schematically depicts the identification procedure used in this work. Assuming the availability of certain unsprung and sprung natural frequencies from an experiment, as well as of some basic material and geometry parameters, the proposed procedure is capable of identifying the unknown sample stiffness.

In the first step, the first freely vibrating torsional frequency $T_{1}=\omega_{tor,1}$ (assumed to be measured from an experiment or otherwise available) is used as an input to solve Equation (7) for the nondimensional rotational inertia of the tip ${\hat{I}}_{tor}$. The measured value $T_{1}$ is converted to the nondimensional eigenvalue $\beta_{1}L$ via the relationship given above in the discussion of Equation (7). Then, from Equation (7), we have

(8) $${\hat{I}}_{tor}={\left(\beta_{1}L\right)}^{-1}\cot\left(\beta_{1}L\right).$$

The nondimensional value ${\hat{I}}_{tor}$, once determined, is then converted to the nondimensional value ${\hat{I}}_{t}$ using the relation ${\hat{I}}_{t}={\hat{I}}_{tor}\left[\left(b^{2}+h^{2}\right)/\left(12L^{2}\right)\right]$, where we have used the definitions of these nondimensional quantities and the fact that A=bh and $J=(bh^{3}+hb^{3})/12$ for a rectangular cross section with width *b* and thickness *h*.

Next, ${\hat{I}}_{t}$ and the first freely vibrating unsprung transverse natural frequency $B_{1}=\omega_{1}$ (assumed to be measured from an experiment or otherwise available) are used as input to solve Equation (3) with α=0, ϕ=0, and ℓ=0 for the nondimensional tip mass Δ. Specifically, we find from Equation (3)

(9) $$\Delta =\frac{\left[1+\cos\left(\lambda L\right)\cosh\left(\lambda L\right)\right]-{\hat{I}}_{t}{\left(\lambda L\right)}^{3}\left[\sin\left(\lambda L\right)\cosh\left(\lambda L\right)+\cos\left(\lambda L\right)\sinh\left(\lambda L\right)\right]}{\lambda L\{{\hat{I}}_{t}{\left(\lambda L\right)}^{3}\left[\cos\left(\lambda L\right)\cosh\left(\lambda L\right)-1\right]+\left[\sin\left(\lambda L\right)\cosh\left(\lambda L\right)-\cos\left(\lambda L\right)\sinh\left(\lambda L\right)\right]\}},$$

where λL should be evaluated at the $\lambda_{1}L$ value determined from $B_{1}=\omega_{1}$. The value $B_{1}$ is converted to the nondimensional natural frequency $\lambda_{1}L$ via the relationship given above in the discussion of Equation (3). In the proposed framework, the nondimensional tip length *ℓ* only affects the moment generated by the lateral spring in Equation (2c) and does not influence the rotational inertia of the tip.

Finally, the estimated nondimensional mass Δ and rotational inertia ${\hat{I}}_{t}$ are used, along with the in-contact transverse natural frequency of vibration $B_{1}^{c}=\omega_{1}^{c}$, the lateral to normal stiffness ratio ϕ, and the tip length to cantilever ratio *ℓ* to solve Equation (3) for the nondimensional stiffness of the sample α. Equation (3) can be rearranged into the following quadratic equation in α:

(10) $$c_{2}\alpha^{2}+c_{1}\alpha +c_{0}=0,$$

where the coefficients of this polynomial are

(11a) $$\begin{array}{cc} c_{2}= & 9\ell^{2}\phi \left[\cosh\left(\lambda^{c}L\right)\cos\left(\lambda^{c}L\right)-1\right], \end{array}$$

(11b) $$\begin{array}{cc} c_{1}= & -3\left(\lambda^{c}L\right)[\left({\left(\lambda^{c}L\right)}^{3}\left(\Delta \ell^{2}\phi +{\hat{I}}_{t}\right)\cos\left(\lambda^{c}L\right)+\sin\left(\lambda^{c}L\right)\left(\phi \ell^{2}{\left(\lambda^{c}L\right)}^{2}+1\right)\right)\cosh\left(\lambda^{c}L\right)+\\ \; & \sinh\left(\lambda^{c}L\right)\left(\phi \ell^{2}{\left(\lambda^{c}L\right)}^{2}-1\right)\cos\left(\lambda^{c}L\right)-{\left(\lambda^{c}L\right)}^{3}\left(\Delta \ell^{2}\phi +{\hat{I}}_{t}\right)], \end{array}$$

(11c) $$\begin{array}{cc} c_{0}= & {\left(\lambda^{c}L\right)}^{4}[\left(\left(\Delta {\hat{I}}_{t}{\left(\lambda^{c}L\right)}^{4}-1\right)\cos\left(\lambda^{c}L\right)+\left(\lambda^{c}L\right)\sin\left(\lambda^{c}L\right)\left({\hat{I}}_{t}{\left(\lambda^{c}L\right)}^{2}+\Delta \right)\right)\cosh\left(\lambda^{c}L\right)+\\ \; & \left(\lambda^{c}L\right)\sinh\left(\lambda^{c}L\right)\left({\hat{I}}_{t}{\left(\lambda^{c}L\right)}^{2}-\Delta \right)\cos\left(\lambda^{c}L\right)-\Delta {\hat{I}}_{t}{\left(\lambda^{c}L\right)}^{4}-1]. \end{array}$$

Here, $\lambda^{c}$ represents the in-contact eigenvalue of the problem which is related to $\omega_{1}^{c}$, as described above in the discussion of Equation (3). It should be noted that, in this analysis, we assume that the stiffness ratio ϕ and the length ratio *ℓ* are known, to simplify the estimation procedure. However, Equation (3) could be solved with multiple measured in-contact natural frequencies to provide simultaneous estimates of ϕ, *ℓ*, and α, similar to the approaches discussed in [2,18,19,38].

**Figure 3:**
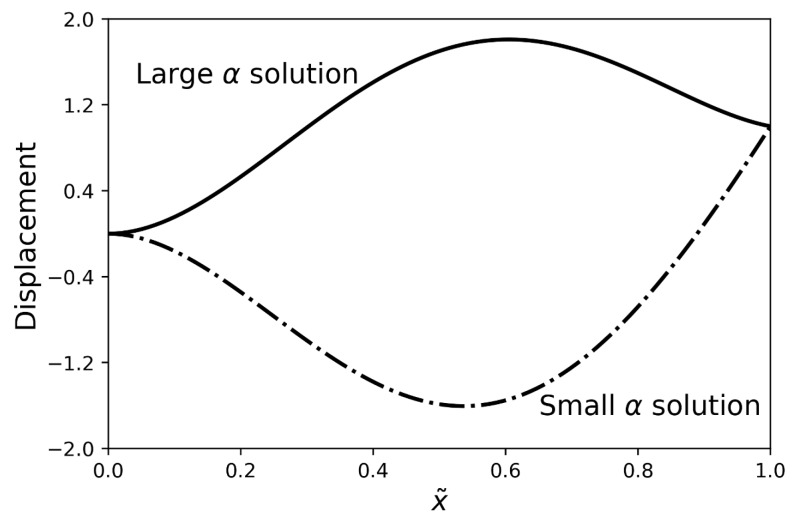
Mode shapes for two distinct α value solutions, at the same frequency, of Equation (10). These solutions represent distinct pairs of *k* and $k^{\prime }$ values. The mode shapes have been normalized such that the tip displacement equals one and the *x*-coordinate has been nondimensionalized by the cantilever length *L* such that $\tilde{x}=x/L$.

When assuming the values of ϕ and *ℓ* and using a single measured in-contact natural frequency to estimate α, situations arise in which two real solutions of α may exist for Equation (10). This occurs when two distinct pairs of *k* and $k^{\prime }$ values, such that $k^{\prime }=\phi k$, satisfy Equation (10) for the same in-contact natural frequency. This apparent paradox is resolved by considering the mode shape of vibration for each solution. For different pairs of *k* and $k^{\prime }$, different mode shapes at the same frequency can satisfy the equations of the system. Figure 3 shows such a case, where the mode shape for the larger α solution is plotted in solid black and the mode shape for the smaller α solution is plotted with a dash-dotted line. It is apparent that the solution for the lower α value is being generated by a higher order mode. Using the mode shape data from the model, along with the knowledge of which specific in-contact natural frequency is being used for property estimation, will ensure the proper α branch selection.

## 3. Numerical Experiments

To verify our identification procedure, in lieu of experimental data on the unsprung and in-contact flexural and torsional vibrations of the prototype cantilever in Figure 1, we conduct numerical experiments to simulate the system vibrational behavior via finite element analysis. A similar approach was previously employed by our group in [39]. The simulations are conducted within the ANSYS Mechanical APDL v. 17 commercial software package. Four different systems are analyzed in detail, as discussed below. The first few modes of vibration are identified for these systems, for a variety of sample stiffnesses. Finite element results on the unsprung flexural and torsional frequencies, as well as on the in-contact flexural frequencies, are then used as input in the identification procedure, as depicted in the flowchart in Figure 2.

The cantilever beam system, schematically depicted in Figure 1, is implemented in the finite element analysis via three-dimensional 2-node beam elements, with six degrees of freedom per node. The beam elements are based on Timoshenko beam theory [36] with shear deformability. Timoshenko beam theory is selected in the numerical experiments as it is expected to accurately model the vibration behavior of the real system. However, since only linear modal analyses are conducted, we do not anticipate significant discrepancies between the Timoshenko and the EB theories for the lowest modes of vibration of sufficiently slender beams.

The sample stiffness during in-contact operation is implemented via one-dimensional linear springs. Note that, as opposed to Figure 1, where we focus on a two-dimensional problem, since this implementation is completely three-dimensional we incorporate two lateral springs $k^{\prime }$ in the *x*- and *y*-directions. The origin of the Cartesian coordinate system coincides with the centroid of the cross section of the fixed end of the beam.

We assume that the cantilever material is silicon with the following properties: E=169GPa, $\rho =2330\;\mathrm{kg}/m^{3}$, and ν=0.25. Similarly, we assume that platinum is used for the tip, with the following material properties: $E_{t}=171\;\mathrm{GPa}$, $\rho_{t}=21,450\;\mathrm{kg}/m^{3}$, and $\nu_{t}=0.39$. Note that, different from our work in Section 2, in our numerical experiment, we assume that the tip is deformable, as it would be in a real AFM scenario. We will comment on the effect of these modeling assumptions in the next section. Throughout the numerical campaign, we set b=30μm and h=2μm for the cross-sectional dimensions of the beam and d=3μm for the diameter of the circular cross section of the tip. We further select $\phi =k^{\prime }/k=0.8$ for the lateral to normal stiffness of the sample. This value is within the theoretically allowed bounds [40,41] and is uniquely determined given the so-called reduced Young’s modulus $E^{*}$ and reduced shear modulus $G^{*}$ of the system. For example, assuming the sample under test is silicon, with the aforementioned properties, and that both tip and sample are comprised of linearly elastic, homogeneous, and isotropic materials, the theoretical value for $\phi =4G^{*}/E^{*}\approx 0.8$.

Four combinations of beam length *L* and tip length $L_{t}$ are explored as reported in Table 1. These combinations are a long cantilever with a long tip (LCLT), a long cantilever with a short tip (LCST), a short cantilever with a long tip (SCLT), and a short cantilever with a short tip (SCST). Table 1 also reports the nomenclature adopted in the rest of the paper as well as the pertinent values of the nondimensional parameter *ℓ*. The table further reports the so-called static and dynamic stiffness ratios, denoted as $R_{s}$ and $R_{d}$, respectively, between the tip stiffness and the cantilever stiffness. Specifically, $R_{s}=k_{c}/k_{t}$, where the tip stiffness is calculated as $k_{t}=3E_{t}I_{t}/L_{t}^{3}$ and represents the ratio of the cantilever to the tip stiffnesses in static conditions. Similarly, $R_{d}=\sqrt{\left(EI\rho_{t}A_{t}L_{t}^{4}\right)/\left(E_{t}I_{t}\rho AL^{4}\right)}$ gives a measure of the overlap between the spectrum of the cantilever and of the tip as if they were independent uncoupled systems. A small or large value of $R_{d}$ indicates essentially decoupled dynamics between the cantilever and the tip. Conversely, $R_{d}\approx 1$ indicates large coupling between the two. In the ideal case of a rigid tip, we have $R_{s}=0$ and $R_{d}=0$. Thus, the larger the corresponding numbers in Table 1, the further the departure from the initial hypotheses of a rigid tip. However, significant numerical departure from these values do not necessarily indicate poor predictions, as explained later.

The cantilever and the tip are meshed with beam elements with a uniform length of 0.1μm. For the shortest tip length considered in this study $L_{t}=10\;\mu$m, this choice still leaves 100 elements along the axis of the tip (and significantly more along the axis of the beam), which is deemed satisfactory to capture the first few structural modes of the system. Since we are interested in the first flexural and the first torsional frequency, for each simulation case, we extract the lowest 10 structural modes. Although the exact ordering in the spectrum of flexural, torsional, and other out-of-plane vibrations depends on the particular geometric configuration, as well as on the values of the sample stiffness, the frequencies $B_{1}$, $T_{1}$, and $B_{1}^{c}$ that are used as input in our model are always within the first ten modes and are thus available from the simulations.

Simulations are conducted for the four geometries for α=0, representative of the unsprung case as well as for values of α spanning the values $\left[10^{-3},10^{3}\right]$, thus capturing a broad range of sample stiffnesses, from very soft to very stiff, when compared to the static cantilever stiffness $k_{c}$.

## 4. Results and Discussion

### 4.1. Parameter Identification

Table 2 shows the predictions of the nondimensional added mass Δ and rotational inertia ${\hat{I}}_{t}$ for the four cantilever cases tested using the estimation method depicted in Figure 2. In this table, we also report values for the added mass parameter calculated as if the tip were a point mass $m_{t}^{\prime }=\rho_{t}A_{t}L_{t}$, and the rotational inertia parameter calculated as if the tip were a rigid rod pinned at one of its ends, so that $I_{t}^{\prime }=m_{t}L_{t}^{2}/3$. Note that $m_{t}^{\prime }$ and $I_{t}^{\prime }$ are, in general, different from the values used in the boundary conditions in Equation (2) and lead to nondimensional parameters, respectively indicated in Table 2 as $\Delta^{\prime }$ and rotational inertia ${\hat{I}}_{t}^{\prime }$. These parameters are defined as

(12a) $$\begin{array}{cc} \; & \Delta^{\prime }=\frac{m_{t}^{\prime }}{m_{c}}=\frac{\rho_{t}A_{t}L_{t}}{\rho AL}, \end{array}$$

(12b) $$\begin{array}{cc} \; & {\hat{I}}_{t}^{\prime }=\frac{I_{t}^{\prime }}{\rho AL^{3}}=\frac{m_{t}L_{t}^{2}/3}{\rho AL^{3}}. \end{array}$$

It is important to observe that the estimated nondimensional parameters are point-mass and point-inertia representations of the physical tip. The physical tip has spatial dimensions and inherent flexibility. Poor agreement of the estimated values with the prediction from Equation (12) does not necessarily indicate poor model performance. Specifically, for the Δ determinations, the discrepancy between the values estimated and the values determined with Equation (12a) are within 10% of each other for the long tip cases, but are very different for the short tip cases. Similarly, while discrepancies on the ${\hat{I}}_{t}$ determinations are within approximately 30% for the long tip cases, negative values are, surprisingly, observed for ${\hat{I}}_{t}$ for the short tip cases. This behavior is likely due to the model trying to capture unmodeled effects caused by the dynamics of the tip that, in simulations, can lead to negative values for ${\hat{I}}_{tor}$ in Equation (8).

**Figure 4:**
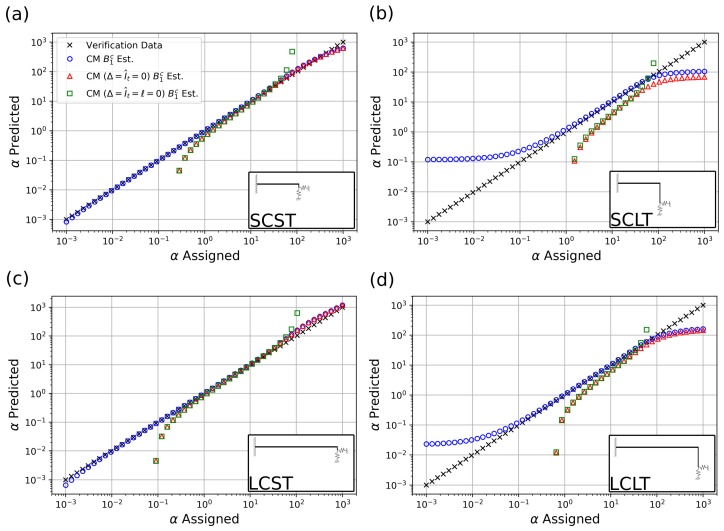
Model predictions versus FEA assigned values (verification data) for the four cantilever geometries in Table 1. Panels (**a**–**d**) correspond to different cantilever/tip geometries indicated by the inset figures. Black *x*’s represent the prescribed verification data, blue circles represent the current model proposed in this work, red triangles represent the current model with no added mass and no rotational inertia, and green squares represent the current model with no added mass, no rotational inertia, and zero tip length.

**Figure 5:**
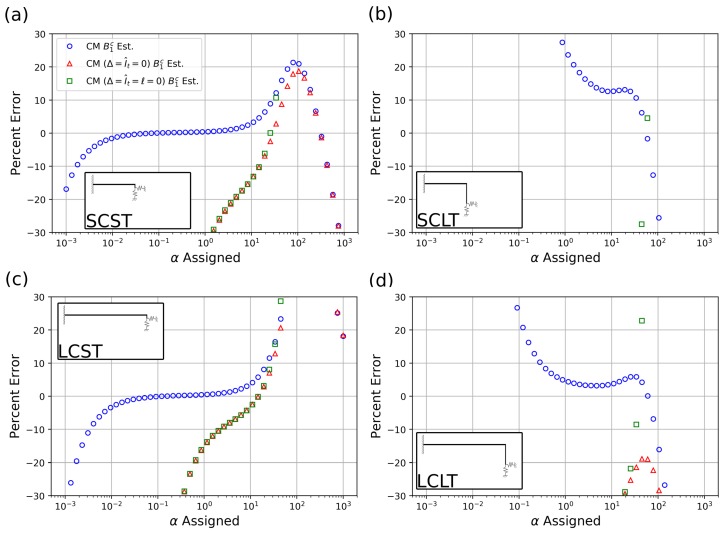
Percent error of the model predictions. Panels (**a**–**d**) correspond to different cantilever/tip geometries indicated by the inset figures.

Figure 4 shows the model results of the estimation of the nondimensional stiffness α versus the assigned values of α used in the FEA simulations discussed in Section 3. Blue circles represent results from the current method (CM), as described in Figure 2, which includes the effect of tip length, mass, and rotational inertia. Red triangles represent results of the current method in which added mass and rotational inertia effects are neglected, henceforth referred to as the “massless tip model”. The massless tip model can be found in works such as [18]. Finally, green squares represent the results of the current method in which tip length, mass, and rotational effects are neglected, henceforth referred to as the “no-tip model”. No-tip models can be found in works such as [2,19]. Figure 5 shows the corresponding percentage error of each model relative to the prescribed α values (verification data) for the four explored cantilever geometries in Table 1.

In the discussion of these results, we will first focus on the performance of the current method which incorporates tip length, added mass, and rotational inertia effects. We will then review the effects of neglecting tip length, added mass, and rotational effects.

### 4.2. Detection Range

The characteristic behavior of the current method result curves (blue circles) in Figure 4 can be summarized by observing that, independent of the particular geometry or cantilever case studied, the current method offers accurate detection of the assigned α value in the neighborhood of α=1. The range of accurate estimation varies for every case studied. In particular, we see the emergence of saturation tails at the low- and high-α ranges. The high-α saturation phenomenon was previously observed in a variety of studies, including for example [39], and is similar to the effect of replacing the free end of the cantilever with a simply supported end, as the sample stiffness increases with respect to the cantilever stiffness. That is, after a sufficiently high sample stiffness, the cantilever can no longer detect subsequent increases in stiffness and all higher stiffness can be described by the same fixity condition. Similar to the high-α range, in which the saturation effect is due to the low stiffness of the cantilever system, we posit that in the low-α range, the saturation effect is due to the low stiffness of the sample with respect to the cantilever system.

**Figure 6:**
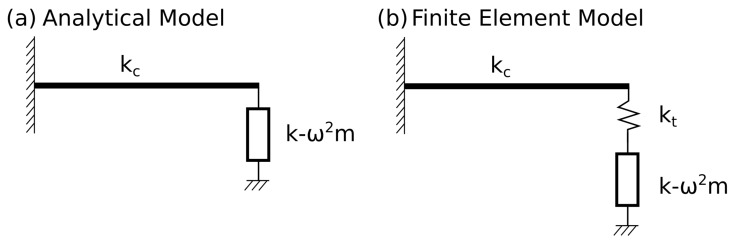
(**a**) Lumped parameter schematic of the current method’s analytical model and (**b**) of the finite element model used for verification.

Thus, the detection range with its two characteristic asymptotic tails, which can be identified as the range of values of α for which the current method yields essentially an “exact” prediction, see also Figure 5, can be interpreted and estimated as follows. In a fundamental sense, and neglecting several second order effects, the current method stipulates that the overall stiffness of the system, as described in Equations (1) and (2), can be described by the stiffness of the cantilever $k_{c}$ in parallel with the normal and tangential (dynamic) stiffnesses $K_{N}$ and $K_{T}$, introduced in the discussion of Equation (2). Neglecting, for simplicity, the contribution of the torsional spring, the overall stiffness of the system can thus be written as $k_{c}+K_{N}$. On the other hand, the finite element model, which for the purpose of this study is a proxy for a real experiment, introduces a slightly more complicated arrangement, whereby the tip stiffness $k_{t}$ is to be considered in series with the (dynamic) stiffness $K_{N}$. The situation is schematically depicted in Figure 6.

Since the proposed model is required to interpret the simulation results within its assumptions, the estimation performance can be understood by equating the analytical model and the simulation stiffnesses, so that

(13) $$k_{c}+\frac{k_{t}K_{N}^{\left(a\right)}}{k_{t}+K_{N}^{\left(a\right)}}=k_{c}+K_{N}^{\left(e\right)},$$

where the superscripts (a) and (e) stand for assigned and estimated, respectively. After some manipulation, and using the definition of $K_{N}$, we have

(14) $$k^{\left(e\right)}=\frac{k_{t}\left(k^{\left(a\right)}-\omega^{2}m_{t}^{\left(a\right)}\right)}{k_{t}+k^{\left(a\right)}-\omega^{2}m_{t}^{\left(a\right)}}+\omega^{2}m_{t}^{\left(e\right)}.$$

This formula allows us to explain the behavior of the estimation performance displayed in Figure 4. First, it is intuitive to assume that the model will yield better predictions as $k_{t}\to \infty$ or, in other words, as $R_{s}\to 0$. Indeed, if $k_{t}$ dominates the denominator of Equation (14), we obtain the ideal case

(15) $$\alpha^{\left(e\right)}\approx \alpha^{\left(a\right)}+\frac{\omega^{2}\left(m_{t}^{\left(e\right)}-m_{t}^{\left(a\right)}\right)}{k_{c}},$$

which shows that the estimated value of α differs from the assigned value of α of a quantity that depends on the tip mass properties estimation error. It can be observed that such error is magnified for larger values of $\omega^{2}/k_{c}$. This indicates that the estimation is expected to be more accurate for relatively stiff cantilevers (“SC” cases) and for shorter tips (“ST”) cases, for which the tip mass $m_{t}$ is small. This is in agreement with what was observed in Figure 4a,c for which, with the tip stiffness being equal, the case SCST displays higher values of $k_{c}$. A secondary effect further complicates this argument, as the quantity $\omega^{2}/k_{c}$ can be presumed to be close to the reciprocal of the system lumped mass *M*. Thus, the estimation is expected to be more accurate for relatively massive systems, which partially explains the better performance of the model for the LCLT case versus the SCLT case, in Figure 4b,d, respectively.

Let us now examine the case where k→∞, in other words, the high-α range, to uncover the reason for the saturation tails. In this case, $k^{\left(a\right)}$ dominates both numerator and denominator of Equation (14), which thus reduces to $k^{\left(e\right)}=k_{t}+\omega^{2}m_{t}^{\left(e\right)}$. Dividing through by $k_{c}$, we obtain

(16) $$\alpha^{\left(e\right)}=R_{s}^{-1}+\omega^{2}m_{t}^{\left(e\right)}/k_{c}.$$

Since $R_{s}\ll 1$, this first term dominates and the asymptotic value of estimated α is equal to $\alpha^{\left(e\right)}=R_{s}^{-1}$. For the four cases depicted in Figure 4, the values of $R_{s}^{-1}$ are approximately 1359 for the SCST case in Figure 4a, 10.9 for the SCLT case in Figure 4b, 10,858 for the LCST case in Figure 4c, and 87 for the LCLT case in Figure 4d. These values also roughly identify the starting point of the asymptotic high-α tails. Indeed, more generally, the saturation tails start occurring for a value of $\alpha^{\left(a\right)}$ roughly equal to $R_{s}^{-1}$. This observation, confirmed by the results in Figure 4, can be simply explained by observing that the horizontal asymptote should begin as $k^{\left(a\right)}$ in the denominator of Equation (14) and becomes comparable in magnitude to $k_{t}$ or, equivalently, when $k^{\left(a\right)}/k_{c}\approx k_{t}/k_{c}$. As expected, model agreement becomes much poorer for the long tip cases (SCLT, LCLT), for which $k_{t}$ is comparatively lower and $R_{s}^{-1}$ is relatively large.

Finally, we examine the case where k→0, that is, the asymptotic horizontal branch in the low-α region. Once again, our point of departure is Equation (14) which, in the limit of zero sample stiffness, upon dividing through by $k_{c}$ reduces to

(17) $$\alpha^{\left(e\right)}=\frac{-\omega^{2}m_{t}^{\left(a\right)}/k_{c}}{1-R_{s}\omega^{2}m_{t}^{\left(a\right)}/k_{c}}+\omega^{2}m^{\left(e\right)}/k_{c}.$$

Note that, in Equation (17), the value of $\alpha^{\left(a\right)}$ does not appear explicitly and, therefore, the model cannot be expected to correctly estimate its value. In the hypothesis of $R_{s}\ll 1$, Equation (17) reduces to $\alpha^{\left(e\right)}\approx \omega^{2}\left(m^{\left(e\right)}-m^{\left(a\right)}\right)/k_{c}$ and, presuming that $\omega^{2}/k_{c}\approx 1/M$ as above, $\alpha^{\left(e\right)}\approx \left(m^{\left(e\right)}-m^{\left(a\right)}\right)/M$. In the “ST” cases, $(m^{\left(e\right)}-m^{\left(a\right)})<0$, as can be appreciated from the values for Δ and $\Delta^{\prime }$ in Table 2. Indeed, for very low values of $\alpha^{\left(a\right)}$, the model yields negative values for $\alpha^{\left(e\right)}$, not displayed in Figure 4. More generally, broader accurate prediction ranges can be expected for the cases with larger $k_{c}$, as the saturation value $\alpha^{\left(e\right)}$ can take on smaller values. This is confirmed in Figure 4, where the SCST case in Figure 4a demonstrates better accuracy at low-$\alpha^{\left(a\right)}$ when compared to the LCST case in Figure 4c. The prominent low-α tails in Figure 4b,d are probably due to the massive tips causing Equation (17) to saturate for moderately large values of $\alpha^{\left(a\right)}$.

While the proposed analysis of the performance of the model is based on simplistic assumptions, our conclusions seem to be justified in view of the numerical experiments. It should also be observed that we have neglected the effect of rotational inertia and rotational stiffness embodied by $K_{T}$ in the derivation of this simple argument. Including the rotational (dynamic) stiffness, however, is not expected to change the qualitative nature of the results.

In addition to the discussion above, in the low-α region, we believe that the large added mass of the tip has the effect of reducing the frequency sensitivity to changes in sample stiffness. Using a one-dimensional approximation of the system, and neglecting the tip stiffness, i.e., presuming $k_{t}\to \infty$, the natural frequency of the system is estimated as $\omega =\sqrt{(k+k_{c})/M}$. The frequency sensitivity to changes in the system stiffness is then given by $d\omega /d(k+k_{c})=1/\left[2\sqrt{M}\sqrt{k+k_{c}}\right]$. Increasing the system mass or stiffness results in a decrease in frequency sensitivity. Thus, we expect the LCLT case, with lower added mass and stiffness, to outperform the SCLT case, as depicted in Figure 4b,d, and Figure 5b,d.

Importantly, model agreement for the SCLT and LCLT cases is also reduced due to the dynamic behavior of the long tip, which can no longer be treated as rigid. For long tips, new models incorporating the dynamics of the tip must be derived. This can be accomplished by considering an explicit EB-type equation for the tip to be coupled with the current governing dynamics in Equation (3). This derivation is however outside the scope of this paper, and will be tackled in subsequent work.

### 4.3. Performance of the Current Model Versus Traditional Models and Outlook

As discussed previously, the current method performs very well within its expected predictive range. For the long cantilever with short tip (LCST) case in Figure 4c, both the massless tip model and the no-tip model accurately predict within ±10% of the assigned α values for assigned α values centered around 10. In fact, their performance is nearly identical for much of the α range. This indicates that added mass and inertia are the primary effects to be considered for much of the α range. At high-α values, the massless tip model performs nearly identically with the current method. This indicates that as α is increased and the free bending mode of the cantilever tends to a highly constrained (or pinned) configuration, the primary effect in this range is due to tip length and not added mass or rotational inertia. Similar performance and behavior of the massless tip and no-tip model can be seen in the short cantilever with short tip (SCST) case in Figure 4a.

In the long tip cases (SCLT and LCLT), both the no-tip and massless tip models perform poorly. The current method performs well for the long cantilever with long tip case (LCLT) for moderate values of α and moderately well for the short cantilever with long tip (SCLT) case for moderate α values. As discussed above, we believe the prediction discrepancies are mainly due to unmodeled tip dynamics.

In the previous discussion, we have assumed perfect knowledge of the system parameters. However, uncertainties in the parameter estimation may exist in a realistic experimental setup. To assess the robustness of the proposed model, we have performed analyses to quantify the effect of uncertainty in the system parameters on the numerical predictions. Table 3 shows these results for one representative assigned $\alpha_{a}$ value, well within the detection range of the model, for the SCST case. In the analyses, we have individually varied each system parameter used in the prediction algorithm by ±10% and calculated the resulting estimation for $\alpha_{e}$ and compared it with the original estimate using the nominal system parameters. For low values of $\alpha_{a}$ ($\alpha_{a}<10$) we see that a ±10% uncertainty in the system parameters has a negligible effect (less than 4%) on the prediction results. The effect of uncertainty in the system parameters increases as $\alpha_{a}$ is increased, especially in the range where model predictions with nominal parameters are already much less accurate. The largest prediction discrepancies are associated with uncertainty in the value of *L*. However, we do not expect difficulties in the experimental determination of this particular parameter within less than 10% uncertainty, for example, via optical microscopy.

Based on the results of this study, in cases where contact resonance microscopy will be used in conjunction with cantilevers that have tips of appreciable length and mass, it is recommended to use the current method for modeling and analysis purposes. It can be appreciated that, even for tips that introduce relatively small added mass, rotational inertia, and tip length effects (see for example the LCST case), the current method extends the predictive α range to very low α values. Thus, the proposed model will be particularly desirable when imaging soft samples in liquids, such as biological materials, using cantilevers with long tips in the trolling mode configuration.

## 5. Conclusions

In this work, we have introduced an updated theoretical model for contact resonance atomic force microscopy, incorporating the effects of a large, massive tip. The model employs a few geometric and material parameters, in conjunction with the knowledge of a limited number of unsprung resonance frequencies for both low-order torsional and flexural modes, for identification of some effective parameter of the system. These identified parameters are then used in the determinations of the in-contact sample stiffness from the knowledge of the in-contact resonance frequencies. The performance of the proposed model has been numerically verified using, in lieu of experimental data, results from high-fidelity finite element simulations. The updated model shows good agreement with the verification data. In general, when performing contact resonance atomic force microscopy using cantilevers with long, massive tips the bending stiffness of the tip should far exceed the bending stiffness of the cantilever. Additionally, the larger the added mass imparted to the cantilever by the tip, the larger the cantilever stiffness should be to ensure accurate measurements.

The model presented in this work has been specifically designed to be simple and easy to use, with only a minimum number of measured parameters to be obtained from an experimental campaign. However, the model is also amenable to several extensions currently in use in the field of contact resonance AFM, which may include incorporating sample viscoelasticity, including an adjustable tip position, and using multiple modes simultaneously for parameter estimation. We expect this model to be the first step in paving the way towards long tip, or trolling mode, configurations of contact resonance atomic force microscopy.

## Figures and Tables

**Table 1:** Geometries explored in the numerical experiments and associated nomenclature, along with resulting values of the nondimensional parameter *ℓ* and of static and dynamic stiffness ratios $R_{s}$ and $R_{d}$, respectively.

Tip Length Beam Length	$L_{t}=10\;\mu$m	$L_{t}=50\;\mu$m
L=150μm	SCST (ℓ=0.067)	SCLT (ℓ=0.333)
	$R_{s}=7.36\times 10^{-4}$	$R_{s}=9.21\times 10^{-2}$
	$R_{d}=7.30\times 10^{-3}$	$R_{d}=1.82\times 10^{-1}$
L=300μm	LCST (ℓ=0.033)	LCLT (ℓ=0.167)
	$R_{s}=9.21\times 10^{-5}$	$R_{s}=1.15\times 10^{-2}$
	$R_{d}=1.82\times 10^{-3}$	$R_{d}=4.56\times 10^{-2}$

**Table 2:** Estimated values of nondimensional added mass and rotational inertia $\left(\Delta ,{\hat{I}}_{t}\right)$ using the method in Figure 2 for the four cantilever cases tested. Nondimensional values $\left(\Delta^{\prime },{\hat{I}}_{t}^{\prime }\right)$ are calculated directly from assigned geometric and material properties from the previous section using Equation (12).

Cantilever	Δ	$\Delta^{\prime }$	${\hat{I}}_{t}$	${\hat{I}}_{t}^{\prime }$
SCST	$5.55\times 10^{-2}$	$7.23\times 10^{-2}$	$-1.30\times 10^{-5}$	$1.07\times 10^{-4}$
SCLT	$3.98\times 10^{-1}$	$3.62\times 10^{-1}$	$2.01\times 10^{-2}$	$1.34\times 10^{-2}$
LCST	$2.10\times 10^{-2}$	$3.62\times 10^{-2}$	$-2.39\times 10^{-5}$	$1.34\times 10^{-5}$
LCLT	$1.66\times 10^{-1}$	$1.81\times 10^{-1}$	$2.28\times 10^{-3}$	$1.67\times 10^{-3}$

**Table 3:** Effect of uncertainty in system parameters on prediction results for one assigned $\alpha_{a}=0.8685$ for the SCST case. The estimation based on nominal system parameters is $\alpha_{e}=0.8720$.

Parameter	Parameter +10% $\alpha_{e}$ Estimate	% Difference Predicted	Parameter −10% $\alpha_{e}$ Estimate	% Difference Predicted
*b*	0.8638	−0.94%	0.8787	0.77%
*t*	0.8675	−0.51%	0.8818	1.13%
*L*	0.9067	3.98%	0.8588	−1.51%
$L_{t}$	0.8709	−0.13%	0.8730	0.12%
ρ	0.8755	0.41%	0.8693	−0.31%
*E*	0.8695	−0.29%	0.8760	0.46%
ν	0.8713	−0.08%	0.8728	0.09%
